# Injectable Stem Cell-Based Therapies for Myocardial Regeneration: A Review of the Literature

**DOI:** 10.3390/jfb16050152

**Published:** 2025-04-23

**Authors:** Marissa Guo, Tatsuya Watanabe, Toshiharu Shinoka

**Affiliations:** 1 Center for Regenerative Medicine, Abigail Wexner Research Institute at Nationwide Children’s Hospital, Columbus, OH 43205, USA; marissa.guo@osumc.edu (M.G.);; 2 Department of Surgery, The Ohio State University Wexner Medical Center, Columbus, OH 43210, USA; 3 Department of Cardiothoracic Surgery, Nationwide Children’s Hospital, Columbus, OH 43205, USA

**Keywords:** cardiac regeneration, stem cell-based therapy, cardiovascular disease, tissue engineering

## Abstract

Stem cell-based therapies are an emerging treatment modality aimed at replenishing lost cardiomyocytes and improving myocardial function after cardiac injury. This review examines the current state of research on injectable stem cell therapies in the setting of cardiovascular disease given their relative simplicity and ability for deep myocardial tissue penetration. Various methods of cell delivery, ranging in level of invasiveness and procedural complexity, have been developed, and numerous cell types have been studied as potential sources of stem cells, each with distinct advantages and disadvantages. We discuss key challenges associated with this approach, including low stem cell retention after transplantation and the innovative biomolecular strategies that have been explored to address this issue. Overall, investigations into the application of stem cells toward cardiac regeneration remain predominantly in the preclinical stage with a number of small, early-phase clinical trials. However, continued scientific advancements in stem cell technology may provide transformative treatment options for patients with heart failure, offering improved survival and quality of life.

## 1. Introduction

In the contemporary era of medicine, cardiovascular disease leads as the primary cause of death and disability worldwide. Ischemic heart disease resulting in the permanent loss of contractile tissue is a main contributor to this morbidity and mortality [1]. Although numerous therapies have been developed with the intent of preventing or alleviating cardiac injury, heart transplantation, which is limited by donor organ scarcity and the risk of immune rejection, remains the only curative treatment option for end-stage heart failure. While there are ongoing advancements in ventricular assist device technology, the risk of infection and thromboembolism continue to be significant issues associated with mechanical circulatory support [2]. Therefore, research on potential techniques to stimulate myocardial regeneration has been a keen area of focus.

The discovery and characterization of cardiac stem cells (CSCs) within the past 20 years has challenged the previously long-held belief that the adult mammalian heart is a terminally differentiated organ with no capability of tissue regeneration after injury [3]. Beltrami et al. were the first to identify self-renewing c-Kit+ cells in the adult heart with the capacity to differentiate into cardiomyocytes (CMs), endothelial cells (ECs), and smooth muscle cells (SMCs) [4]. Nevertheless, the rate of turnover among CMs has been estimated to be as low as 0.45–1% per year [5], which is insufficient to counter the loss of tissue resulting from myocardial infarction (MI). Transplantation of exogenous stem or progenitor cells, however, represents a promising treatment modality with the potential to reverse the pathology underlying heart failure. Stem cell-based therapies for cardiac regeneration have been studied extensively using various cell lines, including embryonic stem cells (ESCs), induced pluripotent stem cells (iPSCs), and mesenchymal stem cells (MSCs) harvested from extracardiac tissues, as sources of functional CMs upon differentiation. Nevertheless, several challenges remain that currently preclude their effective clinical application. Further investigation is needed to discern the ideal timing and approach to delivery for maximal stem cell retention and survival, the optimal combination of stem cells and supporting cells that should be used, and methods of mitigating graft-related complications. Moreover, questions persist regarding the mechanism by which transplanted stem cells induce cardiac tissue healing.

**Figure 1:**
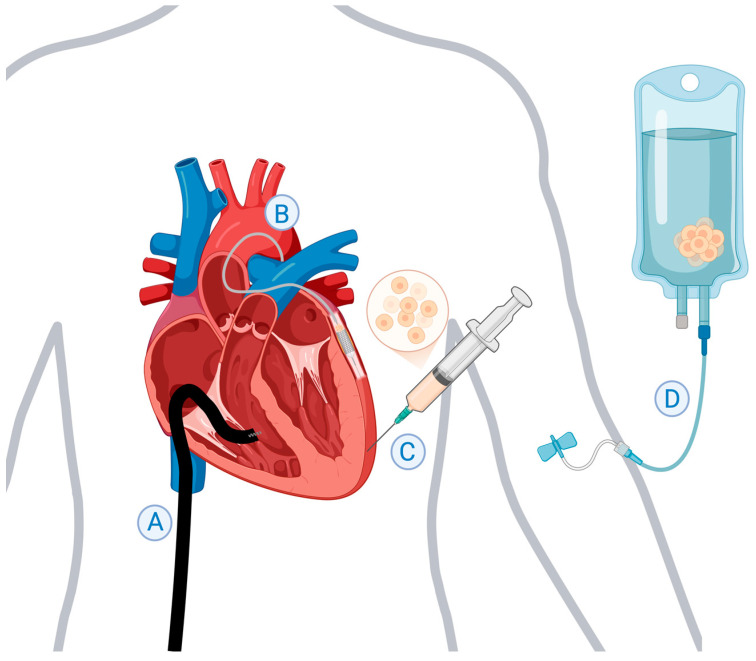
Key methods of stem cell delivery. (**A**) Transendocardial intramyocardial injection performed via cannulation of the femoral vein. (**B**) Intracoronary administration during percutaneous coronary intervention using the stop-flow technique. (**C**) Transepicardial intramyocardial injection performed via sternotomy or lateral thoracotomy. (**D**) Intravenous infusion through a peripheral vein. This figure was created in http://BioRender.com (accessed on 14 April 2025).

This review explores the current state of research on myocardial regeneration through stem cell-based therapies, presenting key methods of delivery (Figure 1), major cell types, recent scientific advancements, and ongoing barriers in translation to widespread clinical applications. Specifically, we focus on injectable approaches to stem cell transplantation. While bioengineered whole tissues offer pre-formed structural support and organization, obstacles associated with this macroscopic approach include difficulties with graft vascularization and limited integration with the host myocardium [6]. In contrast, stem cell injections are simpler and more direct and enable deeper tissue penetration, allowing for a closer examination of the mechanisms involved in stem cell survival and retention, electromechanical coupling, and other signaling interactions with endogenous cells. By examining recent preclinical and clinical studies, our aim is to provide a comprehensive understanding of the value and future prospects of this regenerative treatment strategy.

## 2. Methods of Stem Cell Delivery

### 2.1. Intramyocardial Injection

Direct transepicardial injection into the infarcted myocardium has been the most precise and accurate method of delivery, circumventing the need to address complex issues such as stem cell mobilization and homing. An experiment by Hou et al., using radiolabeled cells, found that among intramyocardial, intracoronary, and retrograde transcoronary venous injections, the direct transepicardial approach yielded the highest rates of MSC retention [7]. However, this procedure is the most invasive and requires entering the chest cavity through a thoracotomy. Clinical trials using this approach are typically performed in patients who are concomitantly undergoing open procedures, such as coronary artery bypass graft surgery (CABG) [8,9,10], although separate operations have been safely performed solely for stem cell injection via lateral mini-thoracotomy [11]. Intramyocardial delivery of stem cells can also be achieved using a catheter-based approach through transendocardial or transvenous intramyocardial injection. These procedures require extensive imaging guidance to properly position the catheter at the site of injection. However, with rapid advancements in imaging modalities and catheterization techniques, the transendocardial approach is currently viewed as a safe and feasible option for stem cell injection. Several clinical trials have been conducted using this method of delivery with minimal risk [12,13,14,15,16,17]. Though less studied, transepicardial injection using a transvenous approach through the coronary venous system has also been shown to be a viable method of depositing stem cells into the myocardium [18,19]. However, navigation through the tortuous venous system can be difficult to accomplish.

### 2.2. Intravenous Infusion

Intravenous infusion is the simplest and least invasive method of stem cell delivery. However, this technique can only be used to treat patients after acute MI, as it is reliant on physiological homing signals to the injured myocardium, which are not present in chronic heart failure. Preclinical studies on swine have shown that intravenous administration of MSCs can lead to augmented vasculogenesis, enhanced regional perfusion, reduced inflammation, and improved ventricular function post-MI [20,21,22]. In humans, a phase I clinical trial demonstrated that intravenous infusions of bone marrow-derived MSCs could be safely performed in patients post-MI, while preliminary data show promising improvements in left ventricular ejection fraction (LVEF) among those in the treatment group [23]. However, other clinical studies investigating stem cell homing found that no stem cells were detectable within the infarcted myocardium after intravenous administration [24,25]. In order to increase the rates of stem cell engraftment and improve the efficacy of intravenous stem cell infusion, scientists have endeavored to develop techniques for homing signal enhancements [26]. For example, a study by Qi et al. showed that an overexpression of colony-stimulating factor 2 receptor β (CSF2Rβ) in MSCs intravenously delivered to mice post-MI led to a significant enhancement in stem cell-mediated proangiogenic, antiapoptotic, and antifibrotic effects [27]. In addition, experiments on rats have shown that multiple repeat intravenous injections were more effective than a single dose of stem cells [28,29].

### 2.3. Intracoronary Administration

Intracoronary arterial injection of stem cells is the most clinically practiced method of cell delivery, especially as it can be performed during percutaneous coronary intervention for the treatment of coronary artery stenosis. With this procedure, selective infusion of stem cells into a coronary artery of interest can allow for the targeted treatment of specific myocardial regions. Starting in 2002, a multitude of clinical trials have been performed evaluating the safety and efficacy of intracoronary stem cell infusion for the treatment of acute MI. However, results from these trials vary widely, with some studies reporting significant improvements in cardiac function associated with stem cell infusion, while others found no differences in outcomes between treatment and control groups [30,31,32,33,34,35,36,37,38]. Of note, intracoronary infusion was found to be a poor method of delivery for CSC spheroids, as the injection of aggregated cells through the left coronary artery led to myocardial scar formation from ischemic injury [39]. The predominant limitation to intracoronary infusion is the inherent difficulty of delivering stem cells to areas with poor perfusion through occluded or stenosed coronary arteries. In patients with arterial occlusion, retrograde stem cell injection through the coronary sinus may be considered. A prospective clinical study by Vicario et al. in patients with chronic refractory angina demonstrated that this form of stem cell delivery was safe and feasible and may have the potential to increase myocardial perfusion and reduce anginal symptoms [40].

### 2.4. Timing of Administration

The optimal timing of stem cell administration requires further elucidation. While the presence of homing signals within the damaged myocardium may favor cell engraftment in the first few days following an acute MI, the hypoxic and nutrient-poor conditions, abundance of free radicals, and adverse inflammation early after reperfusion are deleterious toward transplanted cell survival. Stromal cell-derived factor-1 (SDF-1) has been identified as a crucial chemokine for stem cell homing. In rats, the expression of myocardial SDF-1 was found to peak within the first day following an acute MI and down-trend thereafter, returning to the baseline after the first week [41]. Maximum stem cell recruitment may depend on a balance between SDF-1 expression and recovery of the peri-infarct microenvironment. Studies on rats have indicated that the best outcomes occur when MSCs are given between 7 and 14 days after acute MI in rats [29,42]. In humans, plasma SDF-1, vascular endothelial growth factor A (VEGF-A), and fibroblast growth factor 2 (FGF-2) were found to increase 2 to 3 days after acute MI and remain elevated for up to four weeks [43]. A pooled meta-analysis by Zhang et al. of randomized controlled trials in which patients received intracoronary infusion of BM-MSCs at the time of emergent percutaneous coronary intervention for acute MI found that BM-MSC administration at 4 to 7 days post-MI were superior to that within 24 h in improving contractile function and reducing the need for revascularization [44]. It is possible that the effectiveness of stem cell-based therapies may decrease greater than 14 days post-MI due to the development of irreversible ventricular remodeling and fibrosis.

## 3. Sources of Stem Cells for Differentiation into Cardiomyocytes

### 3.1. Cardiac Stem Cells and Cardiac Progenitor Cells

Cardiac-derived stem and progenitor cells have been studied extensively for their regenerative potential. Their native location and function within the heart, as well as their known capacity to differentiate toward myocardial lineages, make them natural candidates for cell-based therapies for cardiovascular disease. Since the discovery c-Kit+ CSCs in 2001, several different populations of CSCs and cardiac progenitor cells (CPCs) have been identified, including stem cell antigen 1 (Sca-1)+ cells, insulin gene enhancer protein 1 (Isl-1)+ cells, cardiosphere-derived cells (CDCs), cardiac side population cells, epicardial CPCs, and stage-specific embryonic antigen-1 (SSEA-1)+ cells [45]. A meta-analysis of preclinical animal studies evaluating the effect of CSC therapy on cardiac function after MI showed an overall improvement in LVEF [46]. The SCIPIO trial was the first clinical study to evaluate the application of CSC therapy in humans, where patients undergoing CABG received intracoronary infusions of autologous c-Kit+ CSCs harvested from the right atrial appendage. Results show that CSC infusion led to improved left ventricular (LV) function both regionally and globally, reduced the infarct size, and increased tissue viability that persisted up to 1 year after treatment [47]. Of note, reports from this trial were ultimately retracted due to concerns with data integrity [48]. However, similar findings in patients with LV dysfunction months after experiencing an MI were reported from the CADUCEUS trial following intracoronary injection of autologous CDCs obtained from endomyocardial biopsy [49]. Additionally, segmental myocardial function was observed in ALLSTAR, a phase II randomized study investigating the efficacy of intracoronary allogeneic CDC infusion in patients with ischemic cardiomyopathy [50]. The effectiveness of CSC therapy has also been investigated in the setting of congenital heart failure. In a phase II randomized controlled trial called PERSEUS, patients with single ventricle physiology received intracoronary infusions of CDCs after stage 2 or 3 palliation. CDC infusion was found to be associated with improved cardiac function, ventricular volumes, and somatic growth, as well as reduced heart failure status and cardiac fibrosis [51].

### 3.2. Bone Marrow-Derived Mesenchymal Stem Cells (BM-MSCs)

MSCs are multipotent adult stem cells that originate from the mesoderm. MSCs were first discovered in bone marrow, and for many years, bone marrow was the primary source of MSCs for use in regenerative medicine. In 2001, a pioneering study by Orlic et al. revealed that intramyocardial injection of c-Kit+ BM-MSCs in mice following an acute MI produced the formation of new myocytes, ECs, and SMCs upon examination 9 days after cell injection. Furthermore, mice that were treated with BM-MSCs displayed improved LV function and a decreased infarct size compared to control mice [52]. Subsequently, Toma et al. demonstrated that BM-MSCs collected from adult humans were also capable of differentiating into CMs when injected into the LV of immunodeficient mice [53]. Furthermore, a large animal study by Shake et al. showed successful stem cell engraftment with attenuation of contractile dysfunction and adverse LV remodeling associated with intramyocardial injection of autologous BM-MSCs in porcine models of ischemic cardiomyopathy [54].

Several early-phase clinical trials have been conducted to evaluate BM-MSC therapy in patients with ischemic cardiomyopathy, but the published data on its clinical efficacy have been inconsistent. For example, POSEIDON was a randomized comparison of autologous versus allogeneic BM-MSCs delivered by transendocardial injection [12]. While the area of infarct appeared reduced among patients in both arms of the trial, only patients who received autologous BM-MSCs were found to have improved 6 min walk test and Minnesota Living with Health Failure Questionnaire (MLHFQ) scores compared to the baseline. Notably, those who received autologous BM-MSCs experienced a higher incidence of ventricular arrhythmias than those who received allogeneic BM-MSCs. No significant changes in LVEF or maximal oxygen consumption were seen in either treatment group. Meanwhile, the C-CURE trial reported improved LVEF, LV end-systolic volume (LVESV), and 6 min walk test in patients who received BM-MSC therapy compared to those who did not [14]. The TAC-HFT trial also reported improvements in the 6 min walk test, MLHFQ score, and infarct size in patients who received BM-MSC therapy but no differences in the LVEF or LV chamber volume when compared to a placebo [15]. The FOCUS-CCTRN trial noted a slight increase in the mean LVEF but no differences in the LVESV, maximal oxygen consumption, or infarct size [13]. The MSC-HF trial reported improvements in the LVEF, LVESV, stroke volume, myocardial mass, infarct size, and quality-of-life score. Moreover, they observed a significant dose–response effect associated with the number of cells injected [17]. In contrast, both TIME and END-HF trials found no differences in LV function or remodeling among patients who underwent transendocardial injection of mononuclear bone marrow cells (MN-BMCs) compared to those who received a placebo [16,38].

A randomized trial by Zhao et al. reported that transepicardial injection of MN-BMCs in patients undergoing CABG was associated with significant improvements in the New York Heart Association (NYHA) class, LVEF, LV fractional shortening, ventricular wall thickness, and perfusion of ischemic regions [8]. Additionally, the PROMETHEUS trial found that transepicardial injection of BM-MSCs into non-revascularized cardiac segments during CABG resulted in a reduced scar size, improved tissue perfusion, and enhanced regional function [9]. However, randomized trials by Pätilä et al. and Nasseri et al. failed to show any improvements in LV function or remodeling as a result of MN-BMC or BM-MSC injection at the time of CABG [10,55].

### 3.3. Adipose-Derived Stem Cells (ADSCs)

Adipose tissue represents an attractive alternative to bone marrow as a source of MSCs due to its easy accessibility and abundant supply of multipotent adipose-derived stem cells (ADSCs). In 2005, Strem et al. demonstrated that ADSCs were capable of differentiating into CM-like cells expressing cardiac-specific genes in vivo when injected into cryogenically injured myocardium of mice [56]. Yamada et al. showed that intramyocardial injection of ADSCs in rats led to improved contractility and a decreased infarct size after ligation of the left anterior descending artery (LAD). They also observed a differentiation of transplanted ADSCs into CM-like cells, as well as ECs and SMCs [57]. A multitude of other preclinical studies using both small and large animal models have since been published corroborating the beneficial effects of ADSC therapy on cardiac functional recovery, ventricular wall remodeling, and revascularization following ischemic injury [58,59,60,61,62,63,64,65]. Of note, Schenke-Layland et al. and Cai et al. have reported that while they, too, observed improved ventricular function, remodeling, and neovessel formation associated with intramyocardial injection of ADSCs in rats post-MI, these results occurred despite poor long-term donor cell engraftment [66,67]. Meanwhile, Bai et al. found that a small portion of human ADSCs survived at 10 weeks after intramyocardial injection into immunodeficient mice, while only 3.5% of transplanted cells differentiated into CMs or ECs [68].

ATHENA and PRECISE were early clinical trials investigating transendocardial injection of ADSCs in patients with ischemic cardiomyopathy. Participants of the PRECISE trial who received ADSC therapy exhibited higher maximal oxygen consumption levels, a greater LV mass, an improved wall motion score index, and less stress-induced ischemia compared to those who did not [69]. While enrollment in ATHENA was terminated prematurely after two patients experienced a cerebrovascular event that was felt to be unrelated to the trial itself, preliminary results indicate that ADSC therapy was associated with improvements in maximal oxygen consumption, the MLHFQ score, NYHA class, and Canadian Cardiovascular Society (CCS) grade, though no changes in LVEF or LV volumes were observed [23]. No malignant arrhythmias were detected in either trial.

### 3.4. Umbilical Cord-Derived Mesenchymal Stem Cells (UC-MSCs)

Umbilical cord-derived MSCs (UC-MSCs) are another source of MSCs with a few notable advantages over BM-MSCs, such as easier isolation, a higher proliferative capacity, less cellular aging, and the ability for ex vivo expansion with high genomic stability. UC-MSCs can be obtained from different compartments of the umbilical cord, including the cord lining, perivascular tissue, and Wharton’s jelly [70]. Preclinical studies conducted in rodent and porcine models of acute MI have shown that intramyocardial injection of UC-MSCs can result in the preservation of cardiac function, increased vascular density, reduced fibrosis, and decreased apoptosis within the injured tissue [71,72,73]. Such effects are largely attributed to the ability of UC-MSCs to recruit resident CSCs and promote endogenous repair mechanisms. However, a small percentage of transplanted UC-MSCs have been noted to differentiate into CMs and ECs in vivo [71,72]. Similar benefits have been observed in swine following intravenous infusion of UC-MSCs post-MI [22]. RIMECARD was an early randomized controlled trial evaluating intravenous infusion of UC-MSCs in human patients with chronic heart failure. Patients who received UC-MSCs were reported to have experienced significant improvements in LVEF, NYHA class, and MLHFQ score [74].

### 3.5. Amniotic Stem Cells

Amniotic fluid-derived stem cells (AFSCs) and amniotic epithelial cells (AECs) have been investigated as an alternate source of immature stem cells to ESCs with low immunogenicity and anti-inflammatory activity. Moreover, they can be easily obtained in high numbers, which could be especially advantageous for patients diagnosed with congenital heart defects in utero [75]. However, research on their efficacy in cardiac regeneration with direct cell injection for cardiovascular disease has been limited predominantly to rodent studies. Notably, Chiavegato et al. found that xenotransplantation of human AFSCs into rat hearts induced acute rejection regardless of whether the animals were immunosuppressed or immunodeficient, bringing into question the assumption that AFSCs possess tolerogenic properties [76]. Yet, contradictory to these findings, others have reported that injection of human AFSCs or AECs into rat models of MI can lead to stem cell integration and differentiation into CM-like cells that survive up to 2 months post-transplantation [77], which can ultimately result in improved cardiac remodeling and ejection fraction [78,79]. More recent studies have focused on the therapeutic utility of exosomes secreted by AFSCs in place of whole cell injection, citing promising results related to their antiapoptotic, proangiogenic, and antifibrotic effects [80,81].

### 3.6. Embryonic Stem Cells (ESCs)

Embryonic stem cells (ESCs) are pluripotent cells collected from human blastocysts that have the ability to differentiate into cells of all three germ layers. The use of ESCs and their derivatives is faced with many challenges, including ethical concerns, risk for immune rejection, engraftment arrhythmias, genomic instability, and tumorigenic potential. Importantly, ESC populations must undergo a process of purification to isolate cardiovascular progenitor cells and avoid the introduction of undifferentiated ESCs, which leads to the formation of teratomas [82]. In 2005, Ménard et al. conducted a study in which cardiac-committed ESCs from mice were injected into the myocardial scar of sheep two weeks after an induced MI. They found that the mouse ESCs differentiated into CMs capable of proliferating to partially repopulate the scar area. Moreover, both immunosuppressed and non-immunosuppressed sheep experienced improved LV function without teratoma formation or rejection [83]. Other animal studies have shown encouraging results on the ability of transplanted ESC-derived CMs to develop electrical and mechanical coupling with native CMs [84,85,86]. However, the lack of morphologic and functional maturity of ESCs could contribute to the generation of engraftment arrhythmias. Conversely, Laflamme et al. reported in their study that although intramyocardial injection of human ESC (hESC)-derived cardiovascular progenitor cells into infarcted rat hearts attenuated ventricular dilation and led to the preservation of contractile function, the integration of transplanted cells with the host myocardium was limited due to intervening scar tissue [87]. Furthermore, preclinical studies performed in primates using hESC-derived cardiovascular progenitor cells have raised concerns over the sheer number of cells required for injection, as well as the proarrhythmic effect resulting from electromechanical coupling of grafted cells [88,89]. Finally, Zhu et al. observed that none of the hESC-derived cardiovascular progenitor cells injected into the infarcted myocardium of cynomolgus monkeys were present 20 weeks after transplantation, even among primates that were placed on a multi-drug immunosuppressive regimen [90].

Menasché et al. performed the first clinical study investigating the therapeutic potential of ESCs in the setting of ischemic cardiomyopathy. ESC-derived cardiovascular progenitor cells embedded in a fibrin patch were implanted onto the epicardium overlying the area of infarction in six patients with severe LV dysfunction who were concurrently undergoing CABG. The four patients who were assessed at the 1-year follow-up time point were reported to have symptomatic improvements, improved LVEF, and enhanced wall motion of the patched segments. No tumors or arrhythmias were detected postoperatively. Nevertheless, the lack of a control group, small sample size, and simultaneous revascularization at the time of ESC patch implant makes it impossible to draw any conclusions [91].

### 3.7. Induced Pluripotent Stem Cells (iPSCs)

**Figure 2:**
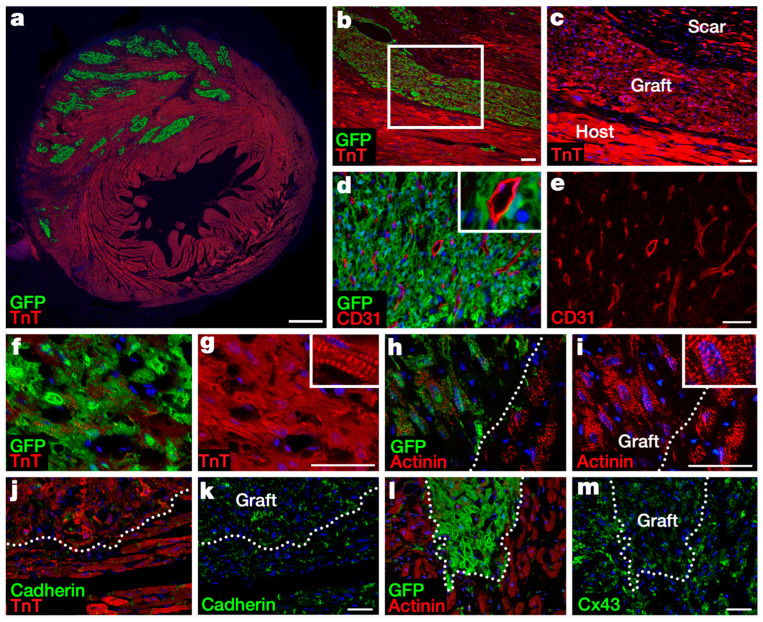
Transplanted iPSC-CMs partially remuscularize infarcted cynomolgus monkey hearts when injected into the myocardium. Grafts were studied on day 84 post-transplantation. (**a**) Grafted GFP^+^ iPSC-CMs (green) survived in the anterior left ventricle. (**b**,**c**) Grafted GFP^+^ iPSC-CMs expressed cardiac troponin T (TnT). (**d**,**e**) Graft cardiomyocytes were well vascularized by host-derived endothelial (GFP^−^/CD31^+^) cells. (**f**–**i**) Graft cardiomyocytes showed sarcomeric structures identified by TnT and ⍺-actinin staining. (**j**–**m**) Cell adhesion and gap junction proteins, cadherin, and connexin 43 (Cx43) were expressed by graft and host cells. Dashed lines indicate the border between graft and host tissues. Nuclei are stained blue. This figure was reprinted with permission from reference [93] copyright 2016 Springer Nature.

Induced pluripotent stem cells (iPSCs) are produced from adult somatic cells through genetic reprogramming. Human iPSC (hiPSC)-derived CMs were shown to be capable of engrafting and proliferating when injected into the infarcted myocardium of immunodeficient mice. Funakoshi et al. observed that transplanted hiPSC-CMs grew substantially within the first month, gradually slowed in proliferation over the next two months, and then lost their proliferation capacity upon maturation thereafter [92]. Shiba et al. reported that allogeneic iPSC-CMs survived for at least 12 weeks and showed electrical coupling with host CMs when injected into the infarcted myocardium of immunosuppressed cynomolgus monkeys (Figure 2). Primates that received iPSC-CMs also exhibited improved contractile function at 4- and 12-weeks post-transplantation, although the incidence of ventricular tachycardia was also transiently increased [93]. To address this issue, Marchiano et al. created hiPSC-CMs that lacked automaticity but contracted when externally stimulated by abolishing depolarization-associated genes, HCN4, CACNA1H, and SLC8A1 and overexpressing hyperpolarization-associated KCNJ2. When injected into healthy porcine hearts, these cells engrafted and coupled electromechanically to host CMs with substantial reduction in arrhythmia burden [94]. Cheng et al. found that co-transplantation of hiPSC-CMs and hiPSC-derived ECs resulted in augmented vascularization and enhanced CM maturity in both mice and rhesus macaques post-MI. Moreover, combined cell therapy led to a larger graft size and decreased rates of arrhythmias in the macaques [95]. While autologous patient-specific iPSCs constitute a desirable source of progenitor cells for cardiac regenerative therapies given their lack of immunogenicity, their use is limited by an expensive and laborious manufacturing process that also precludes their application in acute settings. The development of a universal hypoimmunogenic line of allogeneic hiPSCs through techniques in gene editing has been investigated as a potential solution to this problem [96]. At this time, human iPSC-derived stem cells have not yet reached clinical translation as a treatment for cardiovascular disease.

## 4. Tissue Engineering Strategies to Enhance Cell Engraftment and Survival

Low retention and survival rates post-transplantation represent the principal challenge limiting stem cell-based therapies at this time [97]. Hou et al. found that in porcine models of acute MI, only 11% of transplanted cells remained within the myocardium one hour after intramyocardial injection and <3% of cells remained following intracoronary injection [7]. Meanwhile, Collantes et al. reported cell retention rates of only 13% and 17% following intramyocardial and intracoronary delivery of CSCs in swine with chronic ischemic reperfusion injury. Moreover, they found that none of the cells administered by intracoronary infusion were detectable on histological examination three days later [98]. Strategies that have been explored to augment the longevity of engrafted stem cells include the following: (1) the delivery of cells with scaffolding biomaterials, (2) a combined delivery of multiple cell types or injection of cell aggregates, (3) preconditioning of stem cells prior to transplantation, and (4) genetic manipulation.

### 4.1. Injectable Biomaterials

Injectable polymeric scaffolds can increase cell implantation and survival by providing temporary biomechanical support, as well as enhancing angiogenesis [99]. Hydrogels are hydrated biomaterials that enable three-dimensional encapsulation of living cells and may be composed of various natural and synthetic polymers. Studies on rodent models of acute MI demonstrated significantly higher rates of engraftment when ADSCs were injected with biopolymer scaffolds. Moreover, greater ADSC retention was noted to correlate with improved preservation of cardiac function [100,101]. Wang et al. also observed increased survival and cardiac differentiation when ADSCs were delivered with chitosan components, presumably through the stimulation of collagen synthesis. Rats that underwent intramyocardial injections of ADSCs in chitosan hydrogel had improved cardiac function, a decreased infarct size, and increased angiogenesis compared to those treated with chitosan or ADSCs only [102]. Chitosan hydrogel is also thought to enhance stem cell engraftment by scavenging reactive oxygen species that impair ADSC adhesion molecules, as well as by recruitment of key chemokines for stem cell homing [103]. Similar results were reported by Bai et al. following intramyocardial injections of ADSCs with ECM hydrogels derived from a decellularized heart matrix. Histological examination revealed that ADSCs exhibited higher rates of both engraftment and differentiation when delivered with ECM hydrogel [104]. Takehara et al. demonstrated that hydrogels may be loaded with growth factors and that controlled delivery of these biomolecules can enhance cardiosphere-derived cell engraftment and differentiation in porcine models of chronic MI [105].

### 4.2. Cell Combinations and Aggregates

The combined delivery of multiple cell types may be advantageous, as the actions of one cell type may augment the survival and function of others. Bargehr et al. reported that co-transplantation of hESC-CMs and hESC-derived epicardial cells resulted in an increased graft size, vascularization, and CM proliferation, as well as improved cardiac function, in rats following MI [106]. Ye et al. showed that intramyocardial injection of hiPSC-derived CMs, ECs, and SMCs together led to improved LV function, myocardial metabolism, and arteriole density, as well as reduced apoptosis, infarct size, and ventricular wall stress in swine post-MI. In addition, engrafted cells were found to be present within the myocardium up to four weeks after transplantation [107].

The delivery of stem cell aggregates is another strategy that is thought to have advantages over single-cell suspensions, as cells that are cultured into spheroids possess better three-dimensional cell–cell and cell–matrix interactions. A study by Moon et al. showed that transplantation of hESC-derived CM aggregates resulted in significantly improved cell survival and reduced fibrosis compared to the administration of dissociated cells. In addition, cells delivered in aggregates were noted to be capable of forming gap junctions with the host myocardium [108]. Preclinical experiments in animal models of heart failure by Kawaguchi et al. and Kobayashi et al. have also shown improved stem cell engraftment, long-term benefits to cardiac function, and minimal risk for ventricular arrhythmias after intramyocardial delivery of hiPSC-derived CM spheroids in rats, swine, and cynomolgus monkeys [109,110]. A multicellular approach by Martinez et al. using human UC-MSC aggregates coated with umbilical vein ECs demonstrated that transepicardial injection of these angiogenic spheroids into rat hearts following an MI produced improvements in cardiac function, tissue vascularization, and infarct size [111]. Tan et al. created injectable cardiac organoids comprised of hiPSC-derived CMs, cardiac fibroblasts, ECs, and stromal cells with electrically conductive silicon nanowires to enhance contractile function. They reported that intramyocardial injection of nanowired hiPSC-CM spheroids improved stem cell retention, reduced maladaptive LV remodeling, and accelerated the contractile development of engrafted hiPSC-CMs, thereby eliciting quicker and more significant cardiac functional recovery in rats post-MI [112].

## 5. Tissue Engineering Strategies to Enhance Treatment Efficacy

### 5.1. Stem Cell Preconditioning

Several ex vivo non-genetic manipulation strategies have been explored to enhance stem cell survival and therapeutic activity. Hypoxic preconditioning of stem cells prior to their introduction into the hostile microenvironment of the infarcted host myocardium has been shown to stimulate endogenous cell defense mechanisms and increase the expression of pro-survival and pro-angiogenic factors. Transplantation of MSCs that had undergone hypoxic priming resulted in improved cell survival, angiogenesis, and cardiac function in rodent and porcine models of MI compared to MSCs cultured in normoxic conditions [113,114]. In addition, stem cells may undergo preconditioning with pharmacological agents, cytokines, and growth factors. For example, Tian et al. showed that pretreatment of ADSCs with atorvastatin stimulated the increased expression of CXCR4, which led to the enhanced recruitment of intravenously administered ADSCs to the infarcted myocardium through an upregulation of the SDF-1/CXCR4 signaling pathway in rats. These results were further associated with improvements in cardiac function and remodeling [115]. Similar findings were demonstrated as a result of MSC pretreatment with SDF-1 [116]. Post-MI functional recovery has also been reported to be greater in rats following infusion of MSCs preconditioned with transforming growth factor ⍺ (TGF-⍺) compared with untreated MSCs, presumably through an increase in stem cell VEGF production [117]. Lastly, Cai et al. observed that preconditioning of human CSCs with the heme oxygenase 1 (HO-1) inducer, cobalt protoporphyrin, prior to intramyocardial injection augmented cell proliferation and enhanced LV function and remodeling in mice with ischemic cardiomyopathy [118].

### 5.2. Genetic Modification of Stem Cells

Modulation of stem cell genetics can be accomplished by protein overexpression through deoxyribonucleic acid (DNA) delivery, gene silencing, gene editing, and micro-ribonucleic acid (miRNA)-based modifications. Upregulation of Akt expression has been a well-documented strategy to improve stem cell survival within the infarcted host myocardium. Studies on rat and swine models of MI have demonstrated enhanced cardiac repair and functional recovery in animals injected with Akt-transfected MSCs due to the stimulation of paracrine factor production and the resulting increase in transplanted cell viability [119,120]. Numerous other genes have been implicated in stem cell survival through their anti-apoptotic and pro-angiogenic effects [121,122]. For example, the overexpression of integrin β1 has been shown to increase MSC engraftment through adhesion-mediated cell survival signaling, ultimately leading to improved cardiac performance post-MI [123]. A variety of miRNA targets have also been identified with the potential to enhance the efficacy of stem cell therapy in treating cardiac injury [124]. Li et al. found that transfection of ADSCs with modified mRNA to induce a transient overexpression of vascular endothelial growth factor (VEGF) and basic fibroblast growth factor (bFGF) enhanced the paracrine effect of transplanted cells, leading to reduced ventricular remodeling and enhanced cardiac function [125].

## 6. Stem Cell Mechanism of Action

Initially, the benefits to cardiac function and remodeling following stem cell transplantation were thought to result from tissue regeneration due to donor cell differentiation into new CMs. However, multiple studies have since demonstrated that although stem cell therapy consistently improved function in the infarcted heart, transplanted cells engrafted very poorly or not at all [7,28,98,126,127]. Furthermore, Gnecchi et al. found that functional recovery took place less than 72 h after the injection of BM-MSCs, which was too rapid of a response to be attributed to cardiac regeneration. They also demonstrated that injection of a BM-MSC-conditioned medium led to similar benefits to ventricular contractility and infarct size [128]. Accumulating evidence has instead suggested that the cardioprotective effects associated with stem cell-based therapies arise predominantly from the secretion of paracrine factors that stimulate endogenous repair mechanisms within the host myocardium. The paracrine hypothesis has now been established as the primary means by which stem cells exert their therapeutic impact.

**Figure 3:**
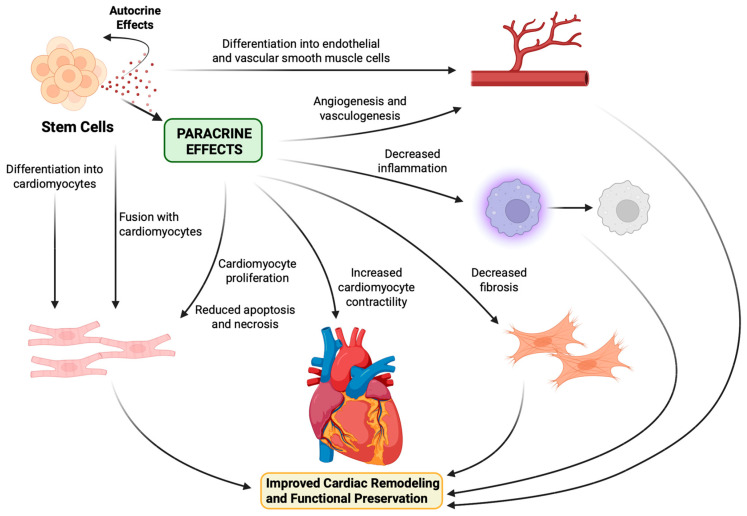
Stem cell mechanisms of cardiac regeneration and repair. This figure was created in http://BioRender.com (accessed on 14 April 2025).

Stem cells release a variety of cytokines, growth factors, miRNA, and other small molecular weight signals to recipient cells within the host tissues that promote neovascularization, while inhibiting inflammation, fibrosis, and cell death (Figure 3) [129,130]. Under hypoxic conditions, MSCs were found to upregulate the expression of several factors, including VEFG, bFGF, hepatocyte growth factor (HGF), insulin-like growth factor 1 (IGF-1), and thymosin β4 (Tβ4), which act to reduce apoptosis and necrosis among surrounding CMs [120,131]. Additionally, Kamihata et al. demonstrated that BM-MNCs injected into ischemic swine hearts increased local cardiac levels of angiogenic ligands, including VEGF, bFGF, angiopoietin-1, and interleukin 1β (IL-1β), and resulting in increased capillary density and regional blood flow [132]. Furthermore, MSC transplantation has been shown to significantly attenuate the increase in cardiac expression of collagen types I and III, tissue inhibitor of metalloproteinase 1 (TIMP-1), and transforming growth factor β (TGF-β) in infarcted rat hearts [133], as well as decrease fibrosis in rat models of dilated cardiomyopathy by reducing the expression of metalloproteinases 2 and 9 [134]. Finally, when injected into the infarcted myocardium of rats, MSCs decreased the expression of pro-inflammatory cytokine tumor necrosis factor ⍺ (TNF-⍺), interleukin 1⍺ (IL-1⍺), and interleukin 6 (IL-6), lessening the degree of LV cavitary dilation and transmural infarct thinning [135]. Interestingly, Tachibana et al. found that hESC- and hiPSC-derived CMs produced higher levels of anti-apoptotic, pro-angiogenic, and pro-cell migratory factors than their pluripotent counterparts, correlating with improved LVEF and myocardial viability when injected into murine models of MI [136].

## 7. Alternative Cell-Free Therapies

### 7.1. Exosome Therapy

With widespread acceptance of the paracrine hypothesis, exosome therapy has emerged as a promising cell-free alternative for the treatment of ischemic heart disease with a relatively simple composition and low immunogenicity. Moreover, exosomes have exhibited exceptional stability and may be stored for extended periods of time, with continuous secretion from immortalized cells ensuring a steady supply [137]. Preclinical studies using both rodent and porcine models have shown improved cardiac function, increased vascularization, and a decreased infarct size following intramyocardial injection of MSC-derived exosomes [138,139,140]. A multitude of factors have been isolated from these secretomes, including VEGF, bFGF, HGF, SDF-1, TGF-β, IGF-1, platelet-derived growth factor (PDGF), and IL-6, as well as an array of miRNAs involved in vascular development, angiogenesis, cell growth, and immunomodulation [141,142]. For instance, Barile et al. demonstrated that extracellular vesicles released by CPCs were enriched in miR-132, which promoted neovascularization and along with miR-210, miR-132, and miR-146a-3p, reduced apoptosis [143]. Others have shown that CPC-derived extracellular vesicles can reduce the inflammation by modulating the expression of pro-inflammatory cytokines, stimulating the release of anti-inflammatory cytokines and inducing macrophage polarization towards the M2 phenotype [144,145].

### 7.2. Direct In Vivo Reprogramming

Direct in situ reprogramming of endogenous non-myocytes into CMs and CPCs represents yet another novel approach to cardiac regeneration that does not rely on the transplantation of exogenous cells and its associated disadvantages [146,147]. Multiple strategies have been explored, including the regulation of transcription factors, intracellular signaling pathways, microRNA, and the epigenetic landscape. Both viral- and nanomaterial-based methods have been used to deliver reprogramming vectors [146]. For instance, Wang et al. developed a non-viral biomimetic system comprised of mesoporous silicon nanoparticles coated with FH peptide-modified neutrophil-mimicking membranes and loaded with miR-1, miR-133, miR-208, and miR-499. Intravenous administration of these nanoparticles successfully resulted in the reprogramming of fibroblasts into CM-like cells, improving cardiac function and attenuating fibrosis, in murine models of MI [148]. While numerous studies have demonstrated successful tests in vivo, research into direct reprogramming is still early, and further investigation is needed to establish more standardized protocols, ensuring safe and consistent results, before moving towards clinical translation.

## 8. Comment

With modern advancements in stem cell research and bioengineering, stem cell-based therapies could represent the next revolutionary step to the treatment of cardiovascular disease given their tentative ability to reverse heart failure pathology and restore cardiac function. As a relatively simple and direct approach, injectable cell therapies have been studied extensively while investigating the regenerative potential of transplanted stem cells in the injured myocardium. Additionally, cell injection carries the distinct advantage of ensuring deep tissue penetration. Multiple methods of cell delivery, ranging in level of invasiveness and procedural complexity, have been developed, and numerous cell types have been explored as possible sources of stem cells, each with individual benefits and shortcomings. Nevertheless, several challenges persist that prevent injectable stem cell-based therapies from reaching more widespread clinical application.

The current state of stem cell research for cardiac regeneration remains predominantly in the preclinical stage, though a small number of early-phase clinical trials have been conducted. In general, studies diverge considerably in many aspects, such as the type of stem cell used, timing of cell injection, approach to cell delivery, length of follow-up, and methods of evaluating post-treatment outcomes, making it difficult to compare or corroborate findings across the existing literature. For instance, an increasing understanding of the mechanisms underlying stem cell therapies has pinpointed the important contributions of numerous small molecular weight signals, transcription factors, and epigenetic regulators [129,130,146], which brings into question whether certain cell types are more efficacious than others as a result of differences in their molecular profiling [136].

Overall, though animal experiments have tended to demonstrate positive effects following stem cell transplantation, results from clinical trials have been divisive. Notably, clinical studies have differed according to the techniques and metrics by which patient outcomes are evaluated (Table 1). Subjective assessments and findings, such as those measuring quality of life, have varied considerably. Additionally, though the majority of research groups have performed cardiac magnetic resonance imaging (MRI) to examine post-therapy structure and function, many have differed on which measurements are reported. For example, while most studies have utilized LVEF as a measure of cardiac function, a few have shown that in some cases, segmental circumferential strain may improve even when LVEF does not [15,49,50,55]. Finally, stem cell injections in humans are typically performed in conjunction with revascularization procedures, introducing added potential for confounding [8,9,10,30,31,32,33,34,35,36].

Given such conflicting evidence, the true efficacy of injectable stem cell therapies has come under scrutiny. Primarily, low stem cell engraftment and survival rates pose a major limitation. Many injected cells are rapidly cleared from the heart, and those that remain often fail to differentiate into functional CMs and integrate with the host tissue [2,7,68,90,98]. During the acute phase following an ischemic injury, hypoxia and adverse inflammation create a hostile microenvironment that reduces transplanted cell viability [42,113,114]. Meanwhile, the lack of homing signals and the formation of fibrous tissue occurring in the setting of more chronic cardiac disease also limits stem cell retention and effectiveness. Furthermore, long-term cell survival may be impaired by potential immune rejection, and while the use of allogeneic stem cells raises such concerns regarding immunogenicity, the development of autologous cell lines is prohibitively expensive in terms of time, labor, and resources at this time.

A variety of strategies have been investigated to address the issue of low cell retention, ranging from cell preconditioning and genetic modifications to co-injection of biomaterials and supporting cell types [99,107,113,114,118,122,125]. Ultimately, direct cell injection may not prove to be the most effective use of stem cells. While very few, if any, transplanted cells contribute to the regeneration of myocardial mass by differentiating into CMs themselves, injected cells are even less likely to organize into the supporting vasculature, collagen skeleton, and electrical conduction system of native cardiac tissue. Stem cells are, however, essential building blocks in the construction of artificial tissues, which represent an alternative approach to cardiac regeneration. Nevertheless, challenges related to adequate vascularization and functional integration with the host myocardium currently pose significant barriers to the clinical use of bioengineered whole tissues [6]. Even so, focus within the field of stem cell research has shifted towards the molecular mechanisms underpinning the therapeutic effect of stem cell transplantation in light of the now widely accepted paracrine hypothesis. Consequently, injectable therapies could still prove to be a valuable treatment modality for heart disease, centering instead on cell-free treatments such as exosome therapy or direct in vivo reprogramming using viral or nanoparticle vectors. Benefits of such strategies include low immunogenicity of the delivered substrate, easily obtained supply, and the potential use of endogenous regenerative mechanisms. Research into these alternative treatment options, at this time, is not yet ready for clinical evaluation. Nonetheless, rapid progress has been made in this field, and the future implementation of stem cell therapies into clinical practice may yet offer a transformative solution for patients with cardiovascular disease and heart failure.

## Figures and Tables

**Table 1:** Characteristics and outcomes of contemporary clinical trials investigating injectable stem cell therapies for ischemic cardiovascular disease.

Author	Year of Publication	Trial Name	Country	Study Design	Experimental Group Sample Size	Cell Origin	Route of Administration		Follow-Up Duration
Zhao et al. [8]	2008	--	China	Phase I RCT	18	Autologous MN-BMCs	Intracoronary infusion during CABG		6 months
Chugh et al. [47] *	2012	SCIPIO	United States	Phase I RCT	20	Autologous CSCs from the right atrial appendage	Intracoronary infusion during CABG		4 and 12 months
Nasseri et al. [55]	2014	Cardio133	Germany	Phase II RCT	30	Autologous BM-MSCs	Intracoronary infusion during CABG		6 months
Karantalis et al. [9]	2014	PROMETHEUS	United States	Phase I RCT	6	Autologous BM-MSCs	Intramyocardial injection during CABG		18 months
Pätilä et al. [10]	2014	--	Finland	Phase II RCT	20	Autologous MN-BMCs	Intramyocardial injection during CABG		12 months
Hare et al. [12]	2012	POSEIDON	United States	Phase I/II RCT	27	Autologous and allogeneic BM-MSCs	Transendocardial injection		13 months
Perin et al. [13]	2012	FOCUS-CCTRN	United States	Phase II RCT	61	Autologous MN-BMCs	Transendocardial injection		6 months
Bartunek et al. [14]	2013	C-CURE	Belgium	Phase I RCT	32	Autologous BM-MSCs	Transendocardial injection		6 months
Santoso et al. [16]	2014	END-HF	Indonesia/Hong Kong	Phase I RCT	19	Autologous MN-BMCs	Transendocardial injection		6 months
Perin et al. [69]	2014	PRECISE	Spain	Phase I RCT	21	Autologous ADSCs	Transendocardial injection		6 and 18 months
Heldman et al. [15]	2014	TAC-HFT	United States	Phase I/II RCT	38	Autologous BM-MSCs and MN-BMCs	Transendocardial injection		12 months
Mathiasen et al. [17]	2020	MSC-HF	Denmark	Phase I RCT	40	Autologous BM-MSCs	Transendocardial injection		12 months
Hare et al. [23]	2009	--	United States	Phase I RCT	39	Allogeneic BM-MSCs	Intravenous infusion		6 months
Bartolucci et al. [74]	2017	RIMECARD	United States	Phase I RCT	15	Allogeneic UC-MSCs	Intravenous infusion		12 months
Strauer et al. [30]	2002	--	Germany	Non-randomized phase I controlled trial	10	Autologous MN-BMCs	Intracoronary infusion via PCI		3 months
Ge et al. [148]	2006	TCT-STAMI	China	Phase I RCT	10	Autologous MN-BMCs	Intracoronary infusion via PCI		6 months
Janssens et al. [147]	2006	--	Belgium	Phase I/II RCT	33	Autologous BM-MSCs	Intracoronary infusion via PCI		4 months
Lunde et al. [31]	2006	ASTAMI	Norway	Phase I/II RCT	47	Autologous MN-BMCs	Intracoronary infusion via PCI		6 months
Assmus et al. [33]	2010	REPAIR-AMI	Germany	Phase II RCT	101	Autologous MN-BMCs	Intracoronary infusion via PCI		24 months
Hirsch et al. [34]	2011	HEBE	Netherlands	Phase II RCT	69	Autologous MN-BMCs	Intracoronary infusion via PCI		4 months
Sürder et al. [35]	2013	SWISS-AMI	Switzerland	Phase II RCT	128	Autologous MN-BMCs	Intracoronary infusion via PCI		4 months
Malliaras et al. [49]	2014	CADUCEUS	United States	Phase I RCT	17	Autologous CDCs obtained from endomyocardial biopsy	Intracoronary infusion via PCI		13.4 months
Wollert et al. [36]	2017	BOOST-2	Germany	Phase II RCT	127	Autologous MN-BMCs	Intracoronary infusion via PCI		6 months
Traverse et al. [38]	2018	TIME	United States	Phase I/II RCT	58	Autologous MN-BMCs	Intracoronary infusion via PCI		24 months
Ostovaneh et al. [50]	2021	ALLSTAR	United States	Phase II RCT	124	Allogeneic CDCs	Intracoronary infusion via PCI		6 months
	**Outcome**								
	**LVEF**	**LVESV**	**Segmental Myocardial Circumferential Strain**	**Infarct Size**	**LV Viable Mass**	**NYHA Class**	**MLHFQ**	**6-Minute Walk Test**	**MVO_2_**
Zhao et al. [8]	↑ compared to control	↓ compared to control	--	--	↑ compared to control	Improved compared to control	--	--	--
Chugh et al. [47] *	↑ compared to baseline	--	--	↓ compared to baseline	↑ compared to baseline	Improved compared to baseline	Improved compared to baseline	--	--
Nasseri et al. [55]	No difference compared to control	No difference compared to control	Improved compared to control (inferior and posterior segments only)	No difference compared to control	--	Worse compared to control	No difference compared to control	No difference compared to control	No difference compared to control
Karantalis et al. [9]	↑ compared to baseline	↓ compared to baseline	No significant change	↓ compared to baseline	↑ compared to baseline	--	--	--	--
Pätilä et al. [10]	No difference compared to control	No difference compared to control	--	↓ compared to baseline	No difference compared to control	--	--	--	--
Hare et al. [12]	No difference compared to baseline	No difference compared to baseline	--	↓ compared to baseline	↑ compared to baseline	No difference from baseline	Improved compared to baseline	Improved compared to baseline	No difference compared to baseline
Perin et al. [13]	↑ compared to baseline	No difference compared to control	--	No difference compared to control	--	Improved compared to baseline	--	--	No difference compared to control
Bartunek et al. [14]	↑ compared to control	↓ compared to control	--	--	--	No difference compared to control	No difference compared to control	Improved compared to control	No difference compared to control
Santoso et al. [16]	No difference compared to control	No difference compared to control	--	No difference compared to control	--	No difference compared to control	--	No difference compared to control	--
Perin et al. [69]	--	--	--	No difference compared to control	↑ compared to baseline	No difference compared to control	--	--	Improved compared to control
Heldman et al. [15]	No difference compared to control	No difference compared to control	Improved compared to control (MSCs only)	↓ compared to control (MSCs only)	↑ compared to control (MSCs only)	No difference compared to control	Improved compared to control (MSCs only)	Improved compared to control (MSCs only)	No difference compared to control
Mathiasen et al. [17]	↑ compared to control	↓ compared to control	--	No difference compared to control	↑ compared to control	No difference compared to control	--	No difference compared to control	--
Hare et al. [23]	↑ compared to baseline, no significant difference compared to control	No difference compared to baseline	--	--	--	--	--	Improved compared to control	--
Bartolucci et al. [74]	No difference compared to control	No difference compared to control	--	--	--	No difference compared to control	No difference compared to control	--	No difference compared to control
Strauer et al. [30]	No difference compared to baseline	↓ compared to baseline	--	↓ compared to control	--	--	--	--	--
Ge et al. [148]	↑ compared to control	--	--	--	--	--	--	--	--
Janssens et al. [147]	No difference compared to control	No difference compared to control	--	No difference compared to control	No difference compared to control	--	--	--	--
Lunde et al. [31]	No difference compared to control	--	--	No difference compared to control	--	--	--	--	--
Assmus et al. [33]	↑ compared to control	No difference compared to control	--	--	--	--	--	--	--
Hirsch et al. [34]	No difference compared to control	No difference compared to control	--	No difference compared to control	No difference compared to control	--	--	--	--
Sürder et al. [35]	No difference compared to control	↓ compared to control (late treatment only)	--	No difference compared to control	No difference compared to control	No difference compared to control	--	--	--
Malliaras et al. [49]	No difference compared to control	No difference compared to control	Improved compared to control	↓ compared to control	↑ compared to control	No difference compared to control	No difference compared to control	No difference compared to baseline	No difference compared to baseline
Wollert et al. [36]	No difference compared to control	No difference compared to control	--	No difference compared to control	--	--	--	--	--
Traverse et al. [38]	No difference compared to control	No difference compared to control	--	↑ compared to control	No difference compared to control	--	--	--	--
Ostovaneh et al. [50]	No difference compared to control	↓ compared to control	Improved compared to control	No difference compared to control	No difference compared to control	--	--	--	--

* This study was ultimately retracted due to concerns over data integrity. Abbreviations: BM-MSCs, bone marrow-derived mesenchymal stem cells; CABG, coronary artery bypass graft surgery; CDCs, cardiosphere-derived cells; CSCs, cardiac stem cells; MN-BMCs, mononuclear bone marrow cells; PCI, percutaneous coronary intervention; RCT, randomized controlled trial; UC-MSCs, umbilical cord-derived mesenchymal stem cells. Abbreviations: LV, left ventricle; LVEF, left ventricular ejection fraction; LVESV, left ventricular end-systolic volume; MLHFQ, Minnesota Living with Heart Failure Questionnaire; MSCs, mesenchymal stem cells; MVO_2_, myocardial volume oxygen; NYHA, New York Heart Association. Symbols: ↑, increased; ↓, decreased; --, not reported.

## Abbreviations

The following abbreviations are used in this manuscript:

ADSC	Adipose-derived stem cell
AEC	Amniotic epithelial cell
AFSC	Amniotic fluid-derived stem cell
bFGF	Basic fibroblast growth factor
BM-MSC	Bone marrow-derived mesenchymal stem cell
CABG	Coronary artery bypass graft surgery
CCS	Canadian Cardiovascular Society
CDC	Cardiosphere-derived cell
CM	Cardiomyocyte
CSF2Rβ	Colony-stimulating factor 2 receptor β
CPC	Cardiac progenitor cell
CSC	Cardiac stem cell
DNA	Deoxyribonucleic acid
EC	Endothelial cell
ESC	Embryonic stem cell
FGF-2	Fibroblast growth factor 2
hESC	Human embryonic stem cell
hESC-CM	Human embryonic stem cell-derived cardiomyocyte
HGF	Hepatocyte growth factor
hiPSC	Human induced pluripotent stem cell
hiPSC-CM	Human induced pluripotent stem cell-derived cardiomyocyte
HO-1	Heme oxygenase 1
IGF-1	Insulin-like growth factor 1
IL-6	Interleukin 6
IL-1⍺	Interleukin 1⍺
IL-1β	Interleukin 1β
iPSC	Induced pluripotent stem cell
iPSC-CM	Induced pluripotent stem cell-derived cardiomyocyte
Isl-1 Insulin	gene enhancer protein 1
LAD	Left anterior descending artery
LV	Left ventricle
LVEF	Left ventricular ejection fraction
LVESV	Left ventricular end-systolic volume
MI	Myocardial Infarction
miRNA	Micro-ribonucleic acid
MLHFQ	Minnesota Living with Heart Failure Questionnaire
MN-BMC	Mononuclear bone marrow cell
MSC	Mesenchymal stem cell
MVO2	Myocardial volume oxygen
NYHA	New York Heart Association
PCI	Percutaneous coronary intervention
PDGF	Platelet-derived growth factor
RCT	Randomized controlled trial
SDF-1	Stromal cell-derived factor 1
SMC	Smooth muscle cell
SSEA-1	Stage-specific embryonic antigen 1
Tβ4	Thymosin β4
TGF-⍺	Transforming growth factor ⍺
TGF-β	Transforming growth factor β
TIMP-1	Tissue inhibitor of metalloproteinase 1
TNF-⍺	Tumor necrosis factor ⍺
UC-MSC	Umbilical cord-derived mesenchymal stem cell
VEGF	Vascular endothelial growth factor
VEGF-A	Vascular endothelial growth factor A
